# Mesenchymal Stem Cell Therapy: Therapeutic Opportunities and Challenges for Diabetic Kidney Disease

**DOI:** 10.3390/ijms251910540

**Published:** 2024-09-30

**Authors:** Jia Cheng, Chun Zhang

**Affiliations:** Department of Nephrology, Union Hospital, Tongji Medical College, Huazhong University of Science and Technology, Wuhan 430000, China; u201712217@hust.edu.cn

**Keywords:** mesenchymal stem cell, diabetic kidney disease, mesenchymal stem cell therapy

## Abstract

Diabetic kidney disease (DKD) is the leading cause of end-stage renal disease (ESRD), which severely affects the quality of patients’ lives. However, the current therapeutic approaches can only postpone its progression to ESRD. It is therefore imperative to develop a novel therapeutic strategy for renal injury in DKD, with the objective of restoring renal function and reversing the process of ESRD. In recent years, the potential of mesenchymal stem cell (MSC) therapy for DKD has garnered increasing attention within the scientific community. Preclinical research on MSC therapy has yielded promising results, and the safety of MSC treatment in vivo has been substantiated in clinical studies. An increasing body of evidence suggests that MSC therapy has significant potential for the treatment of DKD. This article reviews the existing research on MSCs and their derived exosomes in treating DKD and analyzes the underlying mechanism of MSC-based therapy for DKD. Additionally, we discuss the potential of combining MSC therapy with conventional pharmacological treatments, along with the constraints and prospects of MSC therapy for DKD. We hope this review can provide a precise and comprehensive understanding of MSCs for the treatment of DKD.

## 1. Introduction

Diabetic kidney disease (DKD) is the most common microvascular complication of diabetes and the main cause of end-stage renal disease (ESRD) in the majority of countries. Patients with DKD frequently lack efficacious treatment options in the terminal stages and are therefore reliant on hemodialysis or kidney transplantation for survival. The pathogenesis of DKD is a complex process whereby chronic persistent hyperglycemia may lead to nephron damage through immune–inflammatory processes, mitochondrial dysfunction, and oxidative stress [1]. The renal pathological manifestations of DKD patients include mesangial dilation, collagen deposition, basement membrane thickening, podocyte loss and hypertrophy, albuminuria, tubular epithelial atrophy, activated myofibroblasts, matrix accumulation, and inflammatory cell influx [2]. Conventional treatments for DKD are confined to regulating blood pressure (BP), glucose, and lipids, which can merely defer but not wholly reinstate normal renal functioning. It is, therefore, imperative to identify a novel treatment for DKD.

**Figure 1:**
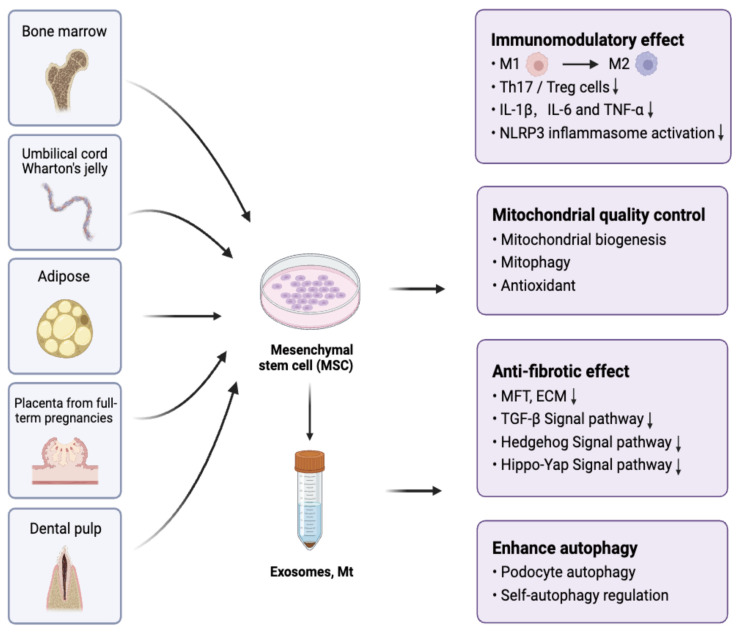
The therapeutic effects of MSCs and their derivatives from different sources in the treatment of DKD. MSCs can be isolated from bone marrow, the umbilical cord, the Wharton’s jelly, adipose tissue, placenta and dental pulp. MSCs and their derivatives have anti-inflammatory and anti-fibrotic effects, control the targeted cells mitochondrial quality, as well as enhance autophagy to protect the kidney from diabetic injury.

Mesenchymal stem cells (MSCs) represent a specific type of undifferentiated stem cell, offering distinctive advantages such as self-renewal capacity, multipotent differentiation potential, and low immunogenicity [3,4]. Furthermore, MSCs possess immunomodulatory [3,5], antioxidant [6], antiapoptotic [7], mitochondrial quality control [8,9], and robust tissue regeneration capabilities [10], rendering them highly versatile in the domain of regenerative medicine. Additionally, MSCs can be sourced from diverse locations, such as bone marrow (BM-MSCs) [11], adipose tissue (ADSCs) [12], umbilical cord (UC-MSCs) [13], Wharton’s jelly (WJMSCs) [14], placenta from full-term pregnancies (PMSCs) [15], and human exfoliated deciduous teeth (SHED) [16] (Figure 1), making them accessible and ethically acceptable for use in research and clinical applications. MSCs can transport intracellular substances, including nucleic acids, proteins, and mitochondria (Mt), through secreting exosomes to damaged cells, thereby regulating the pathological and physiological processes of damaged cells [17,18]. The method of using MSCs and their secreted derivatives (such as exosomes) to treat diseases is collectively called MSC therapy.

MSC therapy has recently garnered significant interest mainly due to its potential for restoring damaged nephrons. There have been notable advancements in the research of MSC therapy in the treatment of DKD. This article provides a summary of the research on MSC therapy in DKD, introduces the potential mechanisms of MSC therapy in DKD treatment, and further analyzes the possibility of combining MSC therapy with traditional pharmacological treatments for DKD. It also considers the prospects and challenges of MSC therapy in treating DKD. This study aims to elucidate the value of MSC therapy in the treatment of DKD and provide new insights into the treatment of DKD.

## 2. The Use of MSC Therapy in DKD

### 2.1. Preclinical Study

The therapeutic potential of MSCs in DKD was initially found because exogenous human bone marrow pluripotent stromal cells (hMSCs) significantly differentiated into endothelial cells in the glomeruli of immunodeficiency NOD/scid diabetic mice [19]. The hMSCs were administered to mice via intracardiac infusion, and subsequent analysis revealed a minor degree of aggregation within the glomeruli. The degree of mesangial thickening was found to be diminished in mice that had been infused with hMSCs, as was the infiltration of macrophages [19]. The author speculated that transplanted hMSCs either prevented pathological changes in the glomeruli or enhanced their regeneration to improve renal lesions in diabetes patients [19]. Subsequently, it was established that MSCs derived from disparate species and sources exhibited therapeutic efficacy in the amelioration of the experimental model of DKD. Concurrently, it was demonstrated that MSCs exert their effects through paracrine pathways [17], specifically by delivering their derivatives such as exosomes [18,20]. Furthermore, it is hypothesized that the mitochondria in MSCs exert a significant influence on the enhancement of mitochondrial quality in damaged renal cells. Derivatives secreted from MSCs are more readily obtainable and storable, with minimal ethical constraints [21], and thus they are regarded as a promising novel therapy for treating kidney injury [22]. The potential of MSC therapy from various sources to treat DKD has been the subject of extensive investigation in recent times. A summary of the preclinical studies (Table 1) of MSC therapy for DKD in the past five years based on species and sources is presented. The studies primarily concentrate on the remission of DKD by BM-MSCs, ADSCs, and UC-MSCs, with a particular focus on UC-MSCs. In contrast to BM-MSCs, the source of UC-MSCs is more readily accessible than bone marrow, obviating the necessity for invasive procedures and significantly reducing the discomfort experienced by patients. Moreover, UC-MSCs exhibit a greater capacity for proliferation [23]. Furthermore, UC-MSCs can be stored at birth and are derived from autologous cells, which exhibit reduced immunogenicity and markedly enhance the safety of mesenchymal therapy. Recently, it was found that MSCs from dental pulp have a similar role in alleviating the progression of DKD and can also be used as a source of materials for future MSC therapy. It is anticipated that in the future, further tissues with robust proliferative and differentiative capabilities will be identified as sources of MSCs.

### 2.2. MSC Therapies Toward Clinical Translation

Currently, a considerable number of clinical studies are investigating the potential of MSC therapy as a treatment for diabetes mellitus (DM). Nevertheless, only two studies have concentrated on the particular issue of kidney damage associated with diabetes and DKD. The clinical application of MSC therapy in the treatment of DKD has made some progress. The two clinical studies primarily explored the safety of bone marrow-derived MSCs (ORBCEL-M) and mesenchymal precursor cells (MPCs, rexlemestrocel-L) in vivo, despite the evidence of adverse effects, in the treatment of DKD [62,63]. Nevertheless, the observation period of rexlemestrocel-L is relatively short, and it cannot be excluded that more rare security incidents may occur after long-term observation. The improvement in renal function reported in the two clinical studies was not statistically significant due to the relatively small sample sizes, although there was a discernible improvement in renal function.

Indeed, the absence of consistent and standardized methodologies to characterize its safety and efficacy represents a significant obstacle to the advancement of MSC therapy toward clinical application. The sources of MSCs are diverse, and there are notable discrepancies in the administration doses employed across disparate studies. It is difficult to determine the optimal efficacious dose in individual clinical studies. Secondly, neither study detected the pharmacokinetics of MSCs in vivo, which is meaningful for us in order to explore effective and safe doses for clinical applications. Indeed, when conducting experiments on cell products, it is essential to confirm the pharmacokinetics of cells within the human body. This will facilitate a deeper comprehension of the metabolism of MSCs in vivo and enable more accurate determination of safe and efficacious doses. The latest research provides some insights into the circulation dynamics of MSCs by detecting the SRY gene in peripheral blood [64]. Furthermore, the relatively small number of subjects included in the two clinical studies, the significant differences in baseline levels observed, and the inconsistent endpoints of observation may have contributed to the lack of significant statistical significance. Given the limited research results on the clinical efficacy, optimal treatment timing, and regimen of MSC therapy for DKD, it is imperative to explore reasonable and safe infusion doses to reach a consensus and standardize clinical trial observation indicators.

MSC therapy in the treatment of DKD has achieved remarkable results in vitro and in vivo. Nevertheless, the proof of human studies has been relatively limited in recent years. There are objective limitations to the translation of animal experiments into human applications. Currently, commonly used DKD animal models include pharmacological induction models (STZ injection) and genetic models (db/db mice). These models cannot well reflect the pathophysiological processes of human DKD. The exact progression of kidney disease may vary greatly from humans, including timing, severity, and specific histopathological features. The timeline of disease progression in animal models is often accelerated compared to humans, leading to a misunderstanding of the long-term effects of interventions. The existing literature has employed unilateral nephrectomy in db/db mouse models as a means of standardizing the onset time of DKD [65]. In addition, human behaviors such as dietary habits, physical activity, and lifestyle choices play a key role in the development of DKD, which cannot be accurately replicated in animal models. It appears that MSCs do not offer a distinct advantage in the treatment of human diabetes nephropathy. There are many challenges in clinical practice, such as heterogeneity in subject renal function and urinary albumin levels, short follow-up observation time, inconsistent biomarker observations, comorbidities, and different drug treatment regimens. These issues collectively contribute to its uncertain clinical value. In the future, it may be necessary to design studies with a larger sample size, that are multicenter, with a longer follow-up time, and with more unified observation endpoints to explore the overall impact of MSC therapy on DKD.

## 3. The Mechanism of MSC Therapy in Treating DKD

### 3.1. Immunomodulatory Effect

MSCs have been demonstrated to possess potent immune regulatory functions. They are capable of ameliorating the local immune microenvironment of the kidney and systemic immune function through direct contact and the secretion of cytokines.

MSCs may play an essential role in suppressing the renal local immune response of DKD by reducing inflammatory cell infiltration and inflammatory response. Studies have indicated that MSCs can suppress the expression of pro-inflammatory factors including IL-1β, IL-6, and TNF-α in the kidney of DKD rats through local immune regulation. Furthermore, the infiltration of inflammatory cells in the kidney tissue of DKD rats treated with MSCs was diminished [28,66], as evidenced by a reduction in CD103 dendritic cells (DCs) and CD8 T cells in the kidney of DKD rats treated with MSC [28]. Additionally, studies have demonstrated that MSC treatment reduces macrophage infiltration by inhibiting the expression of monocyte chemoattractant protein-1 (MCP-1) [66]. The anti-inflammatory effect of M2 macrophages is of pivotal importance in the prevention and treatment of inflammatory diseases. BM-MSCs, UC-MSCs, and their derived exosomes and microRNAs have been demonstrated to enhance macrophage M2 polarization, augment the anti-inflammatory response, and mitigate kidney damage resulting from DKD inflammation [24,41,44,67,68]. MSCs may promote the transformation of macrophages to the M2 phenotype by enhancing the expression of the M2 macrophage marker Arg1 [41], which exerts beneficial effects on mitochondrial dysfunction.

Interestingly, AD-MSCs were demonstrated to increase similar expression of Arg1 and M1 macrophage marker iNOS, resulting in a null effect on mitochondrial dysfunction [41]. The precise reason for this discrepancy in outcomes between the administration of MSCs derived from different sources is currently unclear. The primary immunomodulatory effects of MSC in the kidney are achieved by regulating innate immune cell infiltration, which attenuates local inflammatory responses.

MSCs may also exert therapeutic effects through systemic immune regulation. A single injection of human umbilical cord-derived mesenchymal stem cells (hUC-MSCs) was observed to result in a decrease in serum IL-6, TNF-α, and TGF-β1 levels in the early stage of DKD (8 weeks after STZ injection). In comparison, no change was noted in serum IL-6 and TNF-α levels in the late stage (16 weeks after STZ injection) [19]. This suggests that systemic immune suppression of MSCs is more likely to play a role in the early stage of DKD. The imbalance of TH17/Treg cells plays a vital role in the pathogenesis of diabetes nephropathy. Specifically, the proportion of TH17 cells in the peripheral blood of patients with diabetes nephropathy tends to increase while the proportion of Treg cells decreases. This imbalance may lead to excessive inflammatory response, further exacerbating kidney damage. The infusion of human placenta-derived mesenchymal stem cells (PMSCs) has been demonstrated to promote Th17/Treg balance in the kidneys and blood of DKD rats while simultaneously reducing the levels of pro-inflammatory cytokines (IL-17A and IL-1 β) [58]. It is postulated that MSC may regulate TH17/Treg-related DKD through systemic immune regulation, thereby reducing inflammatory factors in the body and kidneys and weakening the inflammatory response.

In addition, MSCs can inhibit various inflammatory pathways. Human umbilical cord mesenchymal stem cells (UC-MSCs), bone marrow mesenchymal stem cells (BMSCs) [29], and exosomes derived from adipose-derived mesenchymal stem cells (ADMSCs) containing microRNA-26a-5p [36] are beneficial for podocytes and diabetic nephropathy under HG by inhibiting Toll-like receptor (TLR) signaling and suppressing inflammation. MSC-derived exosomes miR-22–3p improve diabetes nephropathy through the NLRP3 signaling pathway [52].

### 3.2. Mitochondrial Quality Control

Many sophisticated quality control mechanisms have been found within mitochondria to counteract stress and maintain the integrity and functionality of organelles, including mitochondrial DNA (mtDNA) repair, mitochondrial dynamics (fusion and fission), mitochondrial autophagy, and mitochondrial biogenesis. The dysregulation of mitochondrial quality control mechanisms may induce mitochondrial damage and dysfunction, leading to cell death, tissue damage, and potential organ failure. DKD is frequently observed to occur alongside a reduction in mitochondrial number and an accompanying impairment in functionality. As a therapeutic method with exogenous restoration of mitochondrial quality control function [69], MSCs are essential in restoring mitochondrial quality in DKD.

It is well-known that MSCs can alleviate high glucose-induced renal mitochondrial dysfunction. Recent studies have shown that MSCs transfer their mitochondria to damaged cells through tunnel nanotubes to improve the mitochondrial quality of target cells. This process involves promoting mitochondrial biogenesis and mitophagy of target cells, which improves the metabolic capacity and antistress ability of cells. Specifically, under co-culture of MSCs and macrophages in vitro, mitochondrial transfer from the former to the latter occurs via tunneling nanotubes (TNT), and high glucose levels can facilitate this process [70]. The mitochondrial function of macrophages was improved by extracting the Mt of MSC and transferring them to macrophages, which may be caused by the activation of PGC-1α-mediated mitochondrial biogenesis and PGC-1α/TFEB-mediated autophagy in macrophages [70]. In vivo and in vitro studies have demonstrated that MSCs can transfer their Mt into diabetes-damaged proximal tubular epithelial cells (PTEC) and glomerular endothelial cells (GECs). MSCs could restore the structure of renal tubules and inhibit cell apoptosis and ROS production by regulating Mt-related factors such as Bcl-2, Bax, and PGC-1 α [26,41]. Moreover, glomeruli’s apoptosis, proliferation, and mitochondrial function have also been improved [31]. The quality of the mitochondria in podocytes plays an integral role in ensuring the proper functioning of these cells. MSCs can maintain the normal structure and function of podocytes by regulating podocyte mitophagy. PMSCs can significantly improve kidney injury and reduce podocyte damage in DKD rats by regulating the SIRT1-PGC-1α-TFAM pathway and enhancing PINK1/Parkin-mediated podocyte mitophagy [57]. Nevertheless, it remains uncertain whether MSCs transfer their mitochondria to podocytes to facilitate podocyte mitophagy and enhance the mitochondrial quality of podocytes. It is currently thought that there are four potential pathways through which Mt can be transferred from MSCs to damaged cells. These include transfer through tunneling nanotubes, gap junctions, cell fusion, microvesicles, and direct uptake of isolated mitochondria [71]. Recent studies have revealed that MSCs may transfer mitochondria to recipient cells through a three-dimensional pathway, as mitochondrial transfer was found in adipose-derived MSCs and breast cancer cells even when the tunneling nanotube (TNT) was blocked [72]. MSCs do not seem to promote the metabolism of recipient cells by exogenously supplementing functional mitochondria. The latest research findings indicate that even dysfunctional mitochondria can trigger mitochondrial autophagy in receptor cells through the typical PINK1/Parkin signaling pathway after internalization and rapidly degrade in autophagosomes. To summarize, exogenous mitochondria serve only as a trigger for autophagy in recipient cells. If it could be demonstrated that mitochondria can be transferred to kidney cells without the involvement of MSCs, this approach would be more ethically sound in clinical applications.

In conclusion, the role of MSCs in alleviating DKD in diabetes may be attributed, at least in part, to the fact that MSCs restore normal cell function by delivering mitochondria to kidney cells or by promoting kidney cells to activate endogenous mitochondrial quality control mechanisms.

### 3.3. Antifibrotic Effect

Fibrosis and epithelial–mesenchymal transition are typical pathological changes associated with DKD, which can result in severe glomerulosclerosis and impaired filtration function. Cytokines, hyperglycemia, and advanced glycation end products (AGEs) have been demonstrated to promote myofibroblast transdifferentiation (MFT) and the profibrotic phenotype of nonfibroblast kidney cells [73]. Renal fibrosis is typified by augmented deposition of the extracellular matrix (ECM), with the degree of renal fibrosis as a predictor of the severity and probability of adverse outcomes in patients [74]. Antifibrotic therapy is of considerable significance in the postponement of the progression of DKD to ESRD.

Multiple MSCs and their derived exosomes have been recognized to play an important role in the delay and reversal of renal fibrosis in diabetic kidney disease by reducing the production of ECM proteins and promoting their degradation [29,34,43,50,55,56,75]. BMSCs reduce the protein expression of plasminogen activator inhibitor-1 (PAI-1) and decrease the accumulation of ECM via inhibiting the TGF-β1/Smad3 pathway, thereby balancing the fibrinolytic system [75]. The paracrine of UC-MSC has been demonstrated to alleviate renal fibrosis in diabetic nephropathy, which is related to the inhibition of the TGF-β1 and Hedgehog signaling pathway. TGF-β-triggered MFT and cell proliferation are mediated by the PI3K/Akt and MAPK signaling pathways. Additionally, the levels of MMP2 and MMP9 increase, resulting in a reduction in fibronectin and type I collagen deposition [50]. Moreover, US-MSCs and their derived exosomes can also inhibit the expression of the crucial protein SMO in Hedgehog signaling, thereby hindering EMT and reducing DN renal fibrosis [56]. It is worth noting that inflammation is also closely related to fibrosis. As mentioned above, the pro-inflammatory factors secreted by macrophages facilitate the MFT and promote the deposition of ECM.

Single-cell RNA transcriptomics has revealed how MSCs-derived small extracellular vesicles (MSCs-EVs) combat DKD-induced fibrosis. It was observed that the administration of MSC sEV induced an increase in the phosphorylation of YAP at serine 381 and serine 127, concomitant with a reduction in the overall protein level of YAP [76], which CK1δ and β-TRCP, the main regulators of YAP degradation, may mediate. When siRNA is used to knock down CK1δ and β-TRCP in MSC sEV, the antifibrosis effect of MSC sEV is attenuated, indicating that MSC sEV exerts its renal protective effect by delivering CK1δ and β-TRCP to the renal tissue [76].

### 3.4. Enhance Autophagy

Autophagy represents a highly conserved lysosomal degradation pathway that removes protein aggregates and damaged organelles to maintain cellular homeostasis. Autophagy is an important stress response mechanism, and its dysfunction has been linked to the pathogenesis of a wide range of diseases. Emerging evidence shows that in individuals with diabetes, an impairment in autophagy may contribute to the development of renal glomerular and tubulointerstitial lesions [77].

MSCs can increase autophagy levels in targeted cells to help maintain normal physiological processes. MSCs can influence the autophagy of immune cells implicated in injury-induced inflammation, thereby reducing their survival, proliferation, and function and facilitating the resolution of inflammation [78]. MSCs have restored renal autophagy throughout the DKD process [49]. MSC-derived exosomes improve diabetes nephropathy by relieving the autophagy attenuation induced by the mTOR signaling pathway [33,37]. Furthermore, MSC-derived exosomes facilitate the degradation of YAP protein in podocytes by enhancing autophagy, thereby reducing its entry into the nucleus and exerting antifibrotic effects [54]. PMSCs alleviate kidney damage in DKD by promoting SIRT1/FOXO1 to encourage autophagy of podocytes [59]. The utilization of the autophagy inhibitors chloroquine and 3-MA (3-methylpurine) resulted in the reversal of the PMSC-mediated enhancement in glucose and lipid metabolism and renal function and the reduction of podocyte damage in DKD [33,59]. Interestingly, the regulation of autophagy in MSCs affects their regenerative therapeutic potential [79].

## 4. The Signaling Pathways Involved in MSC Treatment of DKD

**Figure 2:**
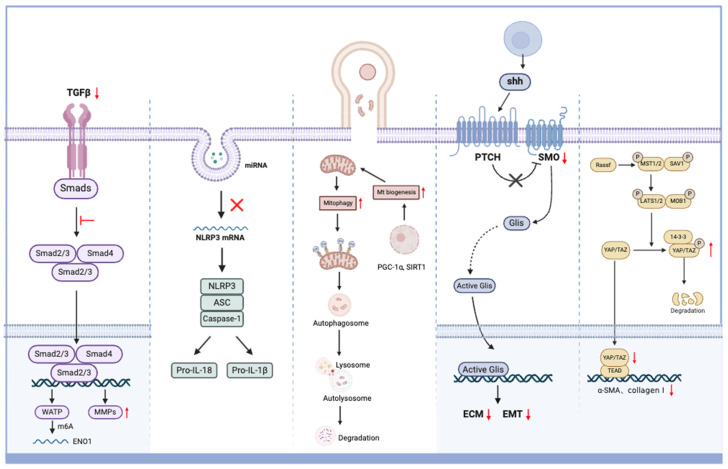
The signal pathways of MSCs in the treatment of DKD. MSCs reduce the expression of TGF-β 1 to inhibit the formation of SMAD complexes, suppress epithelial to mesenchymal transition (EMT), and promote fibrinolysis. MSCs deliver miRNA to receptor cells by secreting exosomes, inhibiting NLPR3 mRNA translation and reducing the release of pro-inflammatory cytokines. MSCs transport mitochondria to receptor cells to activate mitochondrial biogenesis and mitophagy, improving the quality of receptor cell mitochondria. MSCs inhibit the Hedgehog/SMO signaling pathway to reduce the expression of extracellular matrix (ECM) proteins. MSCs inhibit the Hippo/YAP signaling pathway by promoting phosphorylation of YAP protein and reducing ECM protein.

In the past year, there have been many studies that explored the signal pathways involved in the treatment of DKD with MSCs. These studies discussed in detail the multiple signal pathways that MSCs may involve in the treatment of DKD. We summarize the signal pathways that MSC therapy may participate in Figure 2, hoping to provide a new perspective for clarifying the specific mechanism of MSCs in DKD.

## 5. MSC Therapy and Traditional Drug Treatment

Previously, the primary objective in the prevention and treatment of DKD was the strict control of blood glucose and blood pressure. The most widely accepted drugs with renal protective effects are renin–angiotensin–aldosterone system (RAAS) inhibitors such as angiotensin-converting enzyme inhibitors (ACEIs) and angiotensin II receptor blockers (ARBs) and novel hypoglycemics including sodium–glucose co-transporter 2 (SGLT-2) inhibitors and the glucagon-like peptide-1 (GLP-1) receptor agonist. Recently, the new third-generation selective mineralocorticoid receptor antagonist Finerenone, which has great benefits in reducing urinary protein and delaying the risk of end-stage renal disease progression in DKD patients, was developed [80]. However, these symptomatic treatments can only delay kidney damage, with limited therapeutic effects for advanced DKD or ESRD. The loss of nephrons is irreversible. MSC therapy differs from traditional drug therapy because it has enormous therapeutic potential in promoting cell regeneration and restoring normal kidney function. DKD patients often have severe renal dysfunction when seeking medical assistance, and in this sense, MSC therapy has the potential for broader application.

In addition, combining MSC therapy with traditional pharmaceuticals has demonstrated enhanced therapeutic efficacy in the DKD model compared to using either treatment alone. Scientific investigation has established that the conjunction of ADMSCs with the GLP-1 receptor agonist exenatide can markedly enhance renal functionality and mitigate structural alterations by restoring equilibrium in inflammatory, fibrotic, and apoptotic markers [81]. The combination of ADMSCs and the SGLT-2 inhibitor empagliflozin (EMPA) demonstrated superior efficacy in restoring renal function, attenuating renal tubular epithelial cell injury, and reducing podocyte loss in a rat model of DKD compared to a single treatment. The authors posited that ADMSC augmented the renoprotective impact of EMPA by promoting antiapoptotic, anti-inflammatory, and antioxidative stress and restoration of mitochondrial autophagy functions without affecting the hypoglycemic effect of EMPA [82]. UC-MSCs combined with irbesartan significantly exerted better protective effects on glomerular podocyte injury and renal function compared to the administration of MSCs or irbesartan alone [83]. Pre-treating MSCs with drugs and then injecting the treated MSCs into the body also offered certain advantages. Compared with simple MSC treatment, MSCs modified with angiotensin-converting enzyme 2 (ACE2) had better performance in reducing albuminuria and improving glomerulosclerosis. In vitro and in vivo, MSCs-ACE2 is more beneficial than using MSCs alone in reducing Ang II and increasing Ang1-7, thereby inhibiting the adverse effects of Ang II accumulation by downregulating ECM and suppressing the transforming growth factor (TGF-β)/Smad pathway [84]. ACE2 therapy-modified MSCs have additional benefits for the progression of diabetes nephropathy (DN) by inhibiting renal RAS activation and reducing glomerular fibrosis. Nevertheless, the precise nature of the interaction between MSCs and diabetes drugs remains uncertain. In the double-blind control group (placebo) trial of allogeneic BM-MPCs for patients with renal dysfunction due to type 2 diabetes, the subjects also took antidiabetic drugs (including insulin, hypoglycemic traditional medicines such as metformin, and sulfonylureas) while receiving BM-MPCs [62]. However, these subjects did not demonstrate a superior therapeutic effect. Further scientific investigation and larger sample sizes are required to elucidate the impact of MSCs on the efficacy of other hypoglycemics and antihypertensive drugs.

## 6. Challenges and Prospects

Although the beneficial role of MSC therapy in DKD has been widely accepted, some potential issues still need to be improved in its clinical application and promotion.

The safety of MSCs in DKD has been preliminarily verified. To date, few studies have reported adverse events associated with the use of MSCs in DKD. Nevertheless, the safety of this approach remains a topic of debate, given the limited scope of clinical research and the relatively short observation period. A recent study has revealed that MSCs may undergo chromosomal abnormalities even during the early passages of their growth cycle and may develop into malignant tumors if transplanted within the body [85]. DKD-related factors (including hyperglycemia and uremia) may alter the differentiation potential of MSCs, which limits their application. While RNA sequencing indicates that the angiogenic and repair potential of MSCs derived from adipose tissue in DKD subjects can be preserved [86] and that diabetic microenvironment preconditioning of ADSCs has been demonstrated to enhance their antidiabetic, anti-long-term complications and anti-inflammatory effects in type 2 diabetic rats [87], alterations in the transcriptome associated with angiogenesis have been observed [86,88]. Furthermore, a randomized controlled trial yielded evidence that the duration of type 2 diabetes mellitus and obesity influence the efficacy of autologous transferred BM-MSCs [89]. Therefore, the microenvironment of diabetes may result in poor implantation and limited differentiation of stem cells, differentiation into undesired cell lineages, and malignant transformation or genetic distortion of stem cells.

In addition, the MSCs currently used in clinical studies are all administered in a single dose, with limited efficacy. The potential for multiple injections in the future cannot be ruled out, but this may result in additional adverse reactions, including sensitization reactions caused by repeated administrations, local complications (hematoma formation, local infection), and vascular obstruction (dyspnea, oliguria, myocardial infarction, venous thromboembolism events). In the future, if it is demonstrated that repeated in vivo injection of MSCs is safe and well tolerated, repeated administration may prove to be a more efficacious approach and may be suitable for assessing long-term effects on renal function.

Given the potential tumorigenicity and ethical concerns associated with the use of MSCs in clinical practice, there are now more recommendations for cell-free therapies. Cell-free therapy is a biological treatment that primarily uses extracellular matrixs, cytokines, biomolecules, or other biomolecules to treat diseases without the injection or use of live cells. This therapy aims to utilize the natural repair mechanisms within the body or provide bioactive ingredients to promote tissue regeneration and repair. In the treatment of DKD, it has been found that exosomes and mitochondria, two cell products, may have similar effects with applied cells.

As summarized in Table 1, the potential of MSC-exos as a cell-free treatment strategy for DKD has been demonstrated to hold negotiable promises. MSC-exos can avoid many safety issues of MSCs, not only reducing unexpected differentiation of cells at risk of tumor formation but also avoiding secondary ischemic injury caused by vascular coagulation Furthermore, studies have shown that the pretransplantation of exogenous mitochondria into endothelial cells (ECs) induces transient cell protection through mitochondrial autophagy, thereby enabling the implantation of ECs without the necessity of MSC support [69]. These strategies offer novel approaches to transplanting or regenerating DKD glomerular endothelial cells. A detailed exploration of the mechanism of MSCs in the treatment of DKD is likely to facilitate the development of new MSC treatments in the future. Specifically, the extraction and direct input of active ingredients of MSCs can effectively circumvent the potential risks associated with MSC infusion. It is imperative that future studies undertake a comprehensive evaluation of the safety of MSCs in the treatment of DN and that strategies to enhance their safety be explored, including autologous MSC transplantation, immunosuppressive therapy, and gene-editing techniques.

A further challenge is that the biodistribution of MSCs and exosomes after in vivo injection suggested that the aggregation of MSCs in the kidney was limited and their retention time was short, which diminished the therapeutic potential of MSCs in kidney diseases. At present, the most commonly used in vivo MSC injection method is intravenous injection. Still, fluorescence tracing shows that MSCs injected into the tail vein of rats have little or no renal homing. The PMSCs injected into the tail vein of mice mainly accumulated in the thymus and spleen and rarely in the kidney after 24 h in male SD DKD rats [58]. The concentration of human UC-MSCs-EVs in the kidney of db/db mice reached a maximum at 12 h post-tail vein injection, after which the distribution of the EVs in the kidneys decreased, with the majority accumulating in the liver [55]. It is essential to enhance the renal targeted delivery efficiency of MSCs and their derivatives to optimize the utilization rate of MSC therapy [40]. Some scholars have proposed that the direct implantation of mesenchymal cell sheets into the renal capsule can increase the local MSC infiltration rate and demonstrate superior therapeutic efficacy compared to the tail vein injection of MSCs. Stromal cell-derived factor-1 (SDF-1) plays a crucial role in MSC migration, involving activation, mobilization, homing, and retention [90], which may be related to poor homing in DN treatment. In early DN rats, the expression of SDF-1 in the kidneys is weak, which may be the reason for the poor homing effect of transplanted MSCs. The ultrasound-targeted disruption of microbubbles loaded with SDF-1 has the potential to increase the level of SDF-1 in targeted renal tissue, thereby promoting the homing of MSCs to early DN kidneys. However, the impact of this approach on the therapeutic effect on the kidneys has yet to be validated [91,92,93]. Recent studies have explored the combination of MSCs with kidney-targeted materials. Fe3O4-coated polydopamine nanoparticles (NP) internalized MSCs, which enhanced the homing of MSCs transplanted alone in the kidney tissue of DKD mice, improved the accuracy of MSCs homing to the kidneys, and also enhanced the renal function compared to MSCs transplanted alone [94]. It was also reported that the use of a hydrogel containing arginine glycine aspartate (RGD) peptide increased the renal retention and stability of MSC-EVs, had an excellent rescue effect on renal function in the mouse AKI model, reduced histopathological damage and renal tubular damage, and promoted cell proliferation in the early stage of AKI [95]. There is potential for improving the repair efficiency of DKD-damaged cells and enhancing the fibrosis process. Furthermore, three-dimensional (3D) cultured MSCs have been shown to have higher cell survival rates and enhanced anti-inflammatory effects, which may be due to enhanced autophagy of MSCs [79]. In the future, the development of new composite applications of MSCs and related derivatives and materials, as well as the pretreatment of MSCs through various methods, are expected to make new progress in the treatment of DKD.

Despite the potential risks associated with the application of MSCs, their regenerative function in nephrons is irreplaceable, enabling the reversal of damage in advanced kidney disease. In addition, EVs secreted by MSCs have shown great potential as drug carriers in recent years [96,97,98]. Extracellular vesicles offer a promising approach to drug delivery, combining the advantages of cells and nanotechnology in drug delivery. For example, EVs can enhance the stability of drugs, and they have a natural targeting ability based on donor cells when delivering drugs. EVs are nanosized molecules with cell surface substances, so they have biological solid barrier permeability and can selectively penetrate tissue damage. Therefore, EVs will be a promising drug delivery system for treating DKD.

## 7. Conclusions

The management of DKD is a major challenge as there are currently no strategies available to regenerate lost nephron function. The pathological process of DKD is highly complicated, with a variety of contributing factors that collectively impede the development of effective therapies and preclude a complete cure. Therefore, it is important to gain a deeper understanding of the pathological mechanism to facilitate the development of more effective treatments for DKD. The vulnerability and limited regenerative capacity of the nephron significantly impede the recovery of renal function. As a regenerative medicine therapy, MSCs have brought new hope for the treatment of DKD, but various problems derived from them still need to be addressed. Cell-free therapy may be the choice in the future. Exosomes and mitochondria derived from MSCs have great potential in improving renal function. At the same time, the combined application of MSCs and other therapies including MSCs and exosomes as carriers of targeted delivery drugs, MSCs to enhance the efficacy of other therapeutic drugs, and MSCs combined with targeted kidney materials to enhance the efficacy of MSC therapy are worthy of attention. We need to optimize MSC therapy to improve the therapeutic effect in DKD, and further investigation is necessary to elucidate the precise function of MSCs in DKD. Overcoming current challenges will provide a comprehensive rationale for the clinical application of MSC therapy in the treatment of DKD, offering a promising avenue for the clinical management of DKD.

## Figures and Tables

**Table 1:** Preclinical studies on the treatment of DKD with MSCs and their derived exosomes in the past five years.

Type of MSCs	Subjects	Method of DKD Induction	Administration	Frequency and Dose	Effect	Reference
Mouse BM-MSCs	Male BALB/c mice	i.p. of 150 mg/kg STZ	Tail vein injection	Once every two weeks, three times after the onset 5 × 10^5^	MSCs reprogram Mφ into M2 via improvement of the lysosome–autophagy pathway and mitochondrial bioenergetics with transcription factor EB activation.	[24]
	Male mice BTBR.Cg-Lep^ob^/WiscJ	-	Intraperitoneal injection	8th and 10th weeks of age 1 × 10^6^	MSC-treated animals exhibited lighter renal pathological impairment, upregulation of mitochondria-related survival genes, and a decrease in autophagy hyperactivation and apoptosis.	[25]
Rat BM-MSCs	Male C57BL/6 mice male SD rats	i.p. of 150 mg/kg STZ (diabetic mice) tail vein injection of 55 mg/kg of STZ (diabetic rats)	Tail vein injection	8 w and 10 w after the onset 1 × 10^4^	Mt transfer from BM-MSCs to damaged PTECs, injection of BM-MSC-derived isolated Mt in renal capsule share the same effect.	[26]
	Male C57BL/6 mice	Not provided	Tail vein injection	Not provided	MSCs protect DKD kidneys by regulating M6 A methylation through Smad2/3/WTAP/ENO1.	[27]
	Male SD rats	i.p. of 55 mg/kg STZ	Tail vein injection	Once a week for 6 weeks 1 × 10^7^	Show immunosuppression of CD8 T-cell proliferation and activation mediated by CD103 DCs.	[28]
	Male SD rats	i.p. of 65 mg/kg STZ	Tail vein injection	4 × 10^6^	Improve renal function and collagen accumulation; inhibit inflammatory and fibrotic cytokines by downregulating TLR-4/NF-κB expression.	[29]
	Male SD rats	i.p. of 60 mg/kg STZ	Tail vein injection	Once a week for two continuous weeks 5 × 10^6^	MSCs suppress progression of diabetic nephropathy (DN) pathogenesis through LXA4 by targeting TGF-β/Smad signals and pro-inflammatory cytokines in DN.	[30]
	GECs	30 mmol/L D-glucose	Co-culture	5 × 10^5^	Rejuvenate damaged GEs via Mt transfer.	[31]
BM-MSC-Exos	Male SD rats	i.p. of 35 mg/kg STZ	Tail vein injection	Once a week for 12 weeks 100 µg	Increase apoptosis; decrease GLU. Scr, BUN.	[32]
Rat BM-MSCs-derived exosomes	Male albino rats	i.p. of 60 mg/kg STZ	Tail vein injection	Two injections of exosomes 100 μg/kg/dose	Ameliorate diabetic nephropathy by autophagy induction through the mTOR signaling pathway.	[33]
Human BM-MSCs-derived extracellular vesicles (EVs)	Male NSG mice	i.p. of 37 mg/kg STZ for 4 days	Intravenous injection	Once a week for 4 weeks 1 × 10^10^ particles	Revert the progression of glomerular and interstitial fibrosis.	[34]
Rat ADSCs	Male SD rats	8 weeks of high-fat diet (HFD) and a single dose of 25 mg/kg STZ	Tail vein injection	Once a week for 24 weeks 3 × 10^6^	Reduce blood glucose and insulin demand, reduce the expression of SLGT2 of PTEC and reduce kidney damage and inflammation.	[35]
Mouse ADSCs-EVs	C57BL/KsJ db/db	-	Tail vein injection	Once a week for 12 weeks -	miR-26a-5p delivered by ADSC-derived EVs suppress glomerular podocyte apoptosis and protect against DN by regulating TLR4.	[36]
Mouse ADSCs-Exos	C57BL/KsJ db/db	-	Tail vein injection	Once a week for 12 weeks -	Reduce proteinuria, Scr, blood urea nitrogen (BUN), and podocyte apoptosis; miR-486 of ADSCs-Exo promote autophagy flux.	[37]
Rat ADSCs-exos	Male SD rats	i.p. of 60 mg/kg STZ	Tail vein injection	50 μg exosomes t twice a week for 3 weeks	Decrease levels of blood glucose, serum creatinine (Scr), 24 h urinary protein, UACR, and kidney weight/body weight, and they suppressed mesangial hyperplasia and kidney fibrosis, which is related to miR-125a.	[38]
Human ADSCs-EVs	Mouse podocyte clone 5 (MPC5)	Normal glucose (NG, 5.5 mM), NG + mannitol (5.5 mM glucose +24.5 mM MA), HG (30 mM glucose)	-	25 μg/mL	EV-derived miR-15b-5p could protect MPC5 cell apoptosis and inflammation via downregulation of the VEGF/PDK4 axis.	[39]
Rat ADSCs Sheet	Spontaneously Diabetic Torii (SDT) fatty rat	-	Femoral vein injection or renal capsule transplantation	6 × 10^6^	Transplantation of adipose-derived mesenchymal stem cell sheets directly into the kidney improves transplantation efficiency and suppresses inflammation and renal injury progression.	[40]
Human UC-MSCs	Male CD1 mice	i.p. of 80 mg/kg STZ	-	Thrice every 4 weeks after the onset 5 × 10^5^	Promote the expression of Arg1 in macrophages to promote M2 polarization and improve mitochondrial function of renal tubular epithelial cells.	[41]
	Male SD rats	i.p. of 60 mg/kg STZ	Tail vein injection	Once a week for two consecutive weeks 2 × 10^6^	Reduce urinary total protein, UACR, Scr, and BUN, improve renal pathological abnormalities, promote the expression of antiapoptotic protein Bcl-xl, and activate the apoptotic pathway.	[42]
	Male SD rats	i.p. of 60 mg/kg STZ	Tail vein injection	2 × 10^6^	Ameliorate functional parameters, improve renal pathological changes, reduce the levels of pro-inflammatory cytokines (IL-6, IL-1β, and TNF-α) and profibrotic factor (TGF-β) in the kidney and blood.	[43]
	Male SD rats	i.p. of 60 mg/kg STZ	Tail vein injection	Twice; group 1: weeks 7 and 8; group 2: weeks 9 and 10 2 × 10^6^	UC-MSC-derived miR-146a-5p restores renal function in DN rats by facilitating M2 macrophage polarization by targeting TRAF6.	[44]
	Male SD rats	HFD and i.p. of 35 mg/kg STZ	Tail vein injection	Three times every 10 days 2 × 10^6^	Attenuate renal oxidative damage and apoptosis, activate Nrf2.	[45]
	Male SD rats	5/6 nephrectomy, followed by intraperitoneal administration of aminoguanidine (180 mg/kg) and streptozotocin (30 mg/kg)	Intrarenal arterial injection (IRA)	Day 21 after CKD induction 2.1 × 10^5^	IRA injection of xenogeneic MSCs was safe and effectively protected the residual renal function and architectural integrity.	[46]
	Male SD rats	i.p. of 50 mg/kg STZ	Intravenous injection	Once a week for 4 weeks Low-dose hucMSCs-treated group (MSC-L):5 × 10^6^; high-dose hucMSCs-treated group (MSC-H):1 × 10^7^	Improve cell viability, wound healing, and senescence of the high glucose-damaged rat podocytes through a paracrine action mode; activate autophagy and attenuate senescence through the AMPK/mTOR pathway.	[47]
	Male SD rats	HFD for 4 w, followed by i.p. of 30 mg/kg STZ	Tail vein injection	Once per week for four consecutive weeks 1 × 10^7^	HUC-MSCs downregulated the expression of IGF1/IGF1R in the renal tissue of diabetic rats, inhibited the activity of the target genes *CHK2* and *p53*, reduced apoptosis, and improved diabetic nephropathy.	[48]
	Male C57BL/6 mice	i.p. of 60 mg/kg STZ	Tail vein injection	8 w or 16 w after the onset 5 × 10^5^	Reduce uACR and improve multiple glomerular and renal interstitial abnormalities; reduce circulating TGF-β1 and restore intrarenal autophagy; reduce early inflammation of the disease.	[49]
Mouse UC-MSCs	Male BALB/C mice	i.p. of 150 mg/kg STZ	Tail vein injection	Weekly for 4 weeks 1 × 10^4^	Alleviate albuminuria, glomerulus injury, and fibrosis by inhibiting TGF-β1-triggered MFT and cell proliferation mediated by PI3K/Akt and MAPK signaling pathways and elevating the levels of MMP2 and MMP9.	[50]
Human UC-MSCs-exos	Male C57BL/KsJ-db/db	-	Tail vein injection	MSCs: every week for 6 weeks 1 × 10^6^ MSC-exos: twice a week for 6 weeks 10 mg/kg bw	MSC-exos could inhibit high glucose-induced apoptosis and EMT through miR-424-5p targeting of YAP1.	[51]
Human UC-MSCs-exos	Male db/db mice	-	Tail vein injection	3 times in the first week, and then twice a week for the next 3 weeks 100 μg	MSCs-Exos attenuated the expression of inflammatory factors in podocytes and DN mice in vivo and in vitro, inhibited the activation of the NLRP3 signaling pathway, and improved renal injury. MiR-22-3p may play a role in the anti-inflammatory effect of MSCs-Exo.	[52]
Human UC-MSCs-exos	Male C57BL/6J mice; male db/db mice	HFD for 6 weeks, followed by i.p. of 100 mg/kg STZ for three days	Not provided	3 times in the first week, and then twice a week for the next 3 weeks 100 μg	Alleviate the inflammatory response; inhibit the activation of NOD2 signaling pathway; prevent apoptosis; increase cell viability in podocytes.	[53]
Human UC-MSCs-EVs	Male SD rats	HFD and i.p. of 35 mg/kg STZ	Tail vein injection	10 mg/kg	Protein 14-3-3*ζ* in hucMSC-sEVs promotes YAP cytoplasmic retention instead of entering the nucleus, enhancing the level of autophagy in the cytoplasm to remove the excessive YAP protein.	[54]
Human UC-MSCs-EVs	Male C57BLKS/J db/db	-	Intravenous injection	Two times a week from 8 to 18 weeks old 100 ug	Attenuate renal fibrosis and inflammation; MiR-23a-3p and its target Krüppel-like factor 3 (KLF3) inhibit high glucose (HG)-induced STAT3 signaling pathway; miR-23a-3p is packaged into MSC sEV by RNA-binding motif protein X-linked (RBMX).	[55]
Human UC-MSCs/UC-MSCs-exos	Male SD rats	i.p. of 60 mg/kg STZ	Tail vein injection	Twice UC-MSCs: 2 × 10^6^ UC-MSC-exo: 100 μg/kg	Attenuate kidney damage; inhibit EMT and renal fibrosis; decrease SMO expression targeting Hedgehog/SMO pathway.	[56]
Human PMSCs	Male SD rats	i.p. of 60 mg/kg STZ	Tail vein injection	1 × 10^6^	Reverse podocyte injury and mitophagy.	[57]
	Male SD rats	i.p. of 60 mg/kg STZ	Tail vein injection	Once a week for three weeks 1 × 10^6^	Regulate TH17/Treg through systemic immune regulation by upregulating PD-1; enhance the autophagy level of DKD rat kidney and podocyte by upregulating the expression of SIRT1 and FOXO1.	[58,59]
SHED Human BM-MSCs	Male Goto-Kakizaki (GK) rats	HFD for 2–4 weeks	Tail vein injection	4 × 10^6^	Attenuate hyperglycemia, hyperlipidemia, increased urinary albumin excretion, ECM accumulation, and a fractional mesangial area.	[60]
Human Amniotic MSCs	Male SD rats	i.p. of 55 mg/kg STZ	Penile vein injection	2 × 10^6^	Increase the expression of ARF and decrease blood glucose, 24-h urinary protein, Scr, urea, kidney injury molecule-1 (KIM-1), and renal injury index.	[61]
